# Microbial copper reduction method to scavenge anthropogenic radioiodine

**DOI:** 10.1038/srep28113

**Published:** 2016-06-17

**Authors:** Seung Yeop Lee, Ji Young Lee, Je Ho Min, Seung Soo Kim, Min Hoon Baik, Sang Yong Chung, Minhee Lee, Yongjae Lee

**Affiliations:** 1 Korea Atomic Energy Research Institute (KAERI), Daejeon 34057, South Korea; 2 Korean Association for Radiation Application (KARA), Seoul 04790, South Korea; 3 Department of Earth & Environmental Sciences, Pukyong National University, Busan 48513, South Korea; 4 Department of Earth Sciences, Yonsei University, Seoul 03722, South Korea

## Abstract

Unexpected reactor accidents and radioisotope production and consumption have led to a continuous increase in the global-scale contamination of radionuclides. In particular, anthropogenic radioiodine has become critical due to its highly volatile mobilization and recycling in global environments, resulting in widespread, negative impact on nature. We report a novel biostimulant method to effectively scavenge radioiodine that exhibits remarkable selectivity for the highly difficult-to-capture radioiodine of >500-fold over other anions, even under circumneutral pH. We discovered a useful mechanism by which microbially reducible copper (i.e., Cu^2+^ to Cu^+^) acts as a strong binder for iodide-iodide anions to form a crystalline halide salt of CuI that is highly insoluble in wastewater. The biocatalytic crystallization of radioiodine is a promising way to remove radioiodine in a great capacity with robust growth momentum, further ensuring its long-term stability through nuclear I^−^ fixation via microcrystal formation.

The growing amount of radionuclides and radioactive wastes from various nuclear and medical fields is causing public concern due to their short- and long-term radiotoxic impact on nature^1^,^2^,^3^,^4^. In particular, the anthropogenic radioiodine that has been released into nature for decades has become a key issue due to its global recycling^5^,^6^,^7^, which affects the worldwide ecosystem and human health^8^,^9^. Anthropogenic iodine radioisotopes mainly originate from human activities that are performed in the nuclear, industrial, and medical fields^1^,^2^,^3^,^8^,^9^. The radioisotopes are damaging to human health because of their active participation in human metabolic processes. For example, repeated exposure to iodine radioisotopes could lead to metabolic disorders, mental retardation, and thyroid cancer in humans^10^. Large increases of thyroid cancer in children have been reported^11^. In addition, high radioiodine depositions (up to 0.13 MBq/m^2^/month) are also known^6^. As a consequence, the absolute concentrations of radioiodine in some river and lake waters have exceeded pre-anthropogenic levels by several orders of magnitude^12^.

Iodine has only one stable isotope (^127^I) and more than 30 radioisotopes^6^. The iodine radioisotopes are characteristically fast spreading in the global environment (atmosphere, biosphere, and ocean) because they are able to vaporize as I_2_ molecules^13^,^14^ or to freely mobilize as soluble anion species (e.g., I^−^). Soluble iodine species are not easily adsorbed by most minerals and adsorbents because they are strongly excluded from the solid surface by negative repulsion. These features make these iodine species the most difficult isotopes to sequester using conventional methods, such as adsorption technology^15^,^16^,^17^,^18^. In the UNSCEAR-2000 Report^19^, radioiodine is regarded as one of the most difficult radionuclides to treat and manage due to its large quantity and high mobilization.

In recent decades, a large quantity of ^129^I (half-life: 16 million y) from the Sellafield (U.K.) and La Hague (France) nuclear reprocessing facilities has continued to enter the Baltic Sea^20^. Up until 1998, for example, a total of 2300 kg of ^129^I had been discharged into the marine environment by the two European facilities, an amount that is 50 times the total release from nuclear weapon tests^21^. Moreover, the iodine isotopes with short half-life (several min to 60 days) are also problematic to natural environments because of their enormous release and subsequent bioaccumulation^22^. During the global recycling of radioiodine that is released into the environment, it is enriched in seaweed and fish, which are consumed by people for food, with a high accumulation factor (maximum of 10^4^)^23^. The short- and long-term recycling of radioiodine could continue to be a potential risk to humans; thus, a new strategy and new technology are urgent to suppress the gradual widespread bioaccumulation of radioiodine.

## Results and Discussion

**Figure 1:**
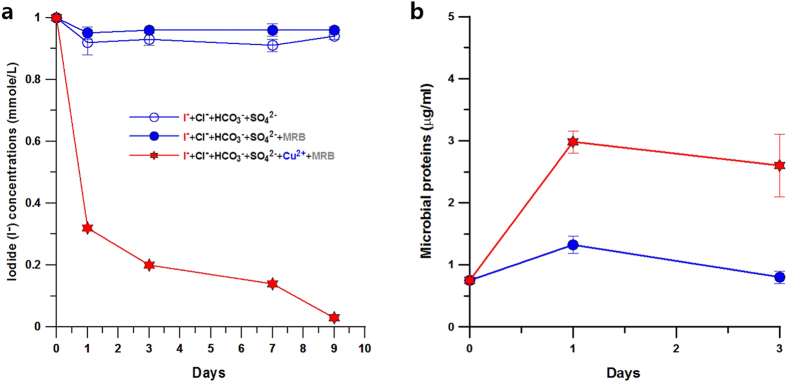
Changes in iodide concentrations and microbial population in anion-rich, anaerobic solutions (pH ~ 7.3). (**a**) Declining behaviours of anionic iodide under different aqueous conditions over time. A rapid decrease in the iodide concentration was observed in a medium with MRB and copper(II). (**b**) MRB growth in the media was monitored with time. A moderate growth of the microbial population was observed in the medium with reducible copper.

Aqueous iodine species usually exist as iodide (I^−^) and iodate (IO_3_^−^), depending on the redox conditions in water^24^. Under a reducing environment or low levels of oxygen, iodine generally exists as I^−^. An experiment for the removal of soluble iodide (I^−^) from anion-rich anaerobic solutions was performed at near-neutral pH (Fig. 1) to determine how background anions (Cl^−^, HCO_3_^−^ and SO_4_^2−^) affect the behaviour of iodide under anaerobic conditions. Soluble iodide is hardly precipitable and capturable without specific adsorbents, regardless of the amount of competitive anions. As expected, there was little decline of the soluble iodide concentrations in the absence of adsorbents (Fig. 1a). However, a decrease of iodide was observed in a medium with bacteria and trace copper, even under highly competitive anion concentrations. The initial iodide concentration (1 mM) quickly dropped to a very low level along with a decrease of the copper concentration (Fig. S1) when the metal-reducing bacteria (MRB) and copper were added to the medium. The mixture of natural common anions did not appreciably interfere with the iodide removal and selectivity. The decrease of iodide concentration contrasted with the increase of the MRB population in the medium (Fig. 1b). Metal-reducing bacteria can obtain energy for growth by electron transport to Cu(II). In general, the metal-reducing microorganisms grow under anaerobic environments by enzymatically coupling the oxidation of organic matter with the reduction of metals^25^,^26^.

**Figure 2:**
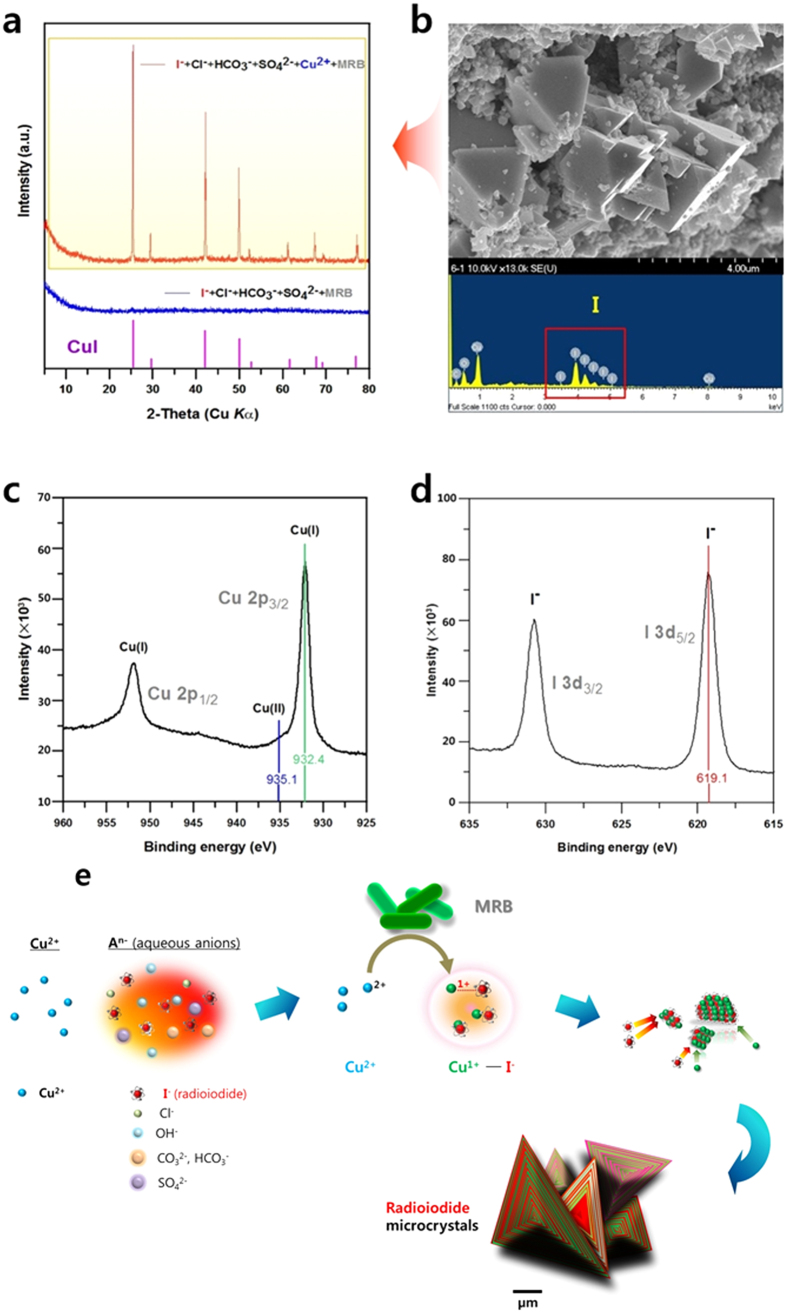
Characterization of the biosynthetic iodide sample and its mineralizing process. (**a**) XRD patterns of the samples that were collected from the media biostimulated with MRB and/or copper. Only in the case of both MRB and copper was the halide mineral (CuI) produced in a nearly pure and highly crystalline form. (**b**) SEM image of the solid sample with microscale crystals of iodide, showing strong EDS peaks of iodine. (**c,d**) High-resolution XPS spectra of the Cu 2p and I 3d regions of the solid sample. (**e**) Schematic of the microbial copper reduction and biogenic growth of radioiodine under competitive conditions.

During the microbially induced Cu reduction, characteristic halide crystals were generated as a solid phase grown to micrometre-sizes, even in the low iodine concentration (i.e., I^−^ ≤ 1 mM). A mineralogical study of the newly formed solid phase was performed by X-ray diffraction (XRD) (Fig. 2a). The powder XRD indicated that the unique biomineralization product derived from the system was cuprous iodide (CuI) crystal. However, other precipitable samples from copper-free media were amorphous, with no characteristic XRD patterns. The scanning electron microscopy (SEM) image shows highly crystalline forms of iodide in Fig. 2b, and its major element (I) is confirmed by the strong EDS peaks. The analytical results shown in Fig. 2a,b reveal that iodide is selectively bound to cuprous Cu^+^, even in the presence of competitive anions. The iodic halide nuclei that formed continued to grow by outcompeting other anions even at room temperature and circumneutral pH (e.g., pH 7.3). In a control experiment (e.g., the loading of Ag^+^ instead of Cu^2+^), however, the crystallization of iodide by combining with silver ions was not largely developed just showing relatively smaller AgI particles with sizes of less than 1.0 μm (Fig. S2).

The XPS analysis of the CuI solid sample shows that the oxidation state for copper was almost +1 (Fig. 2c). The peak at 932.4 eV, which only appeared as Cu(I) after biological Cu(II)-reduction, corresponds to the Cu 2p_3/2_ binding energy of CuI. Furthermore, the presence of anionic iodide (I^−^), as indicated by the I 3d_5/2_ signal at 619.1 eV in Fig. 2d, proves the formation of the CuI crystal phase.

The schematic model for our operating system shown in Fig. 2e exhibits the central role of a biocatalytic process that isolates iodide and allows it to grow in large crystals through a microbial metal reduction^25^,^26^,^27^,^28^. The microbe-driven biological process can be useful to skillfully regulate the mobility of iodide by transforming its aqueous form. A gradual change from water-soluble to insoluble cuprous iodic form may be crucial for the free iodic anions to be restrictive in mobilizing and to join the solid form in water. The continuous and robust bioconversion of copper (Cu^2+^ → Cu^1+^) functions as a driving force to make the CuI nuclei grow to the large solid phase. Copper acts as an important inorganic binder to strongly bond the iodide and iodide ions. As long as the system is sustained, the cuprous iodic nuclei do not remain as halide molecules or colloids but continue to grow into euhedral crystalline solids (~μm) with well-formed faces. The newly formed phase is a very rigid compound, where iodide can be fixed tightly, resulting in its low solubility in water. Because the new phase is extremely insoluble (<0.00042 g/L at 25 °C), it is not perceptibly decomposed in water.

**Figure 3:**
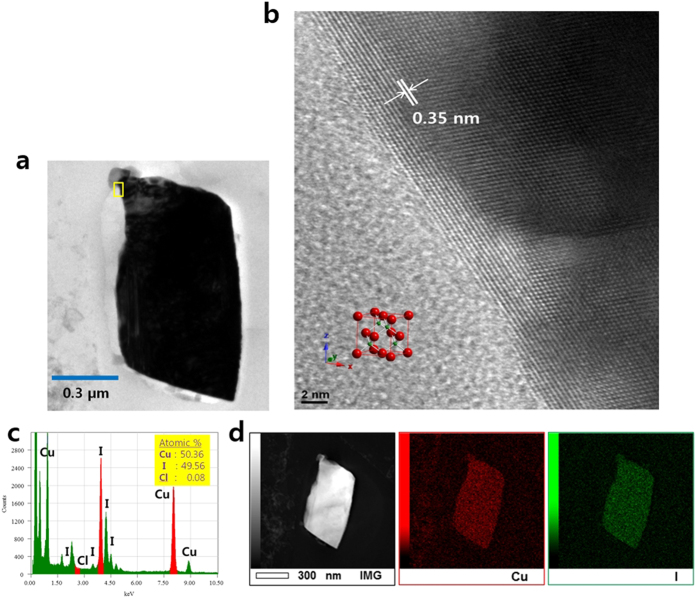
Cross-sectioned HRTEM images of the crystallized iodide sample. (**a**) A thin section (~50 nm) TEM image of the iodide solid sample biosynthesized under the anion-rich system. (**b**) Enlarged view of a small square in (**a**) showing some distinctive lattice fringes (*d* = 0.35 nm) of the crystalline CuI phase. (**c**) TEM-EDS spectrum showing the ratios of the major components. (**d**) Nanoscale probe maps that show homogeneous distributions of copper and iodine in the microcrystal.

As shown in Fig. 3, we microscopically investigated the CuI crystals that were synthesized and grown to micrometre sizes in our biostimulant system. Copper(I) iodide is an inorganic polymer with a zinc-blende structure. Unlike the usual iodide entrapped by common anion-exchangers or adsorbents, the solid phase grew by the continuous attachment of iodide (Fig. 3a). Some specific lattice fringes of the CuI crystal were examined by a high-resolution nanoscale probe and were identified as {111} with a ~0.35-nm-spaced set (Fig. 3b). The TEM-EDS spectrum identified the crystal as almost pure CuI with trace chlorine (0.08 atomic%), demonstrating the higher selectivity of iodine (49.56 atomic%) of >500-fold over chlorine (Fig. 3c). Cl^−^ is unfavourable to be shared by the halide phase due to its lower binding energy with copper. The elemental distribution analysis for the cross-sectioned sample revealed that the iodine component was not locally but homogeneously distributed in the microstructure (Fig. 3d).

**Figure 4:**
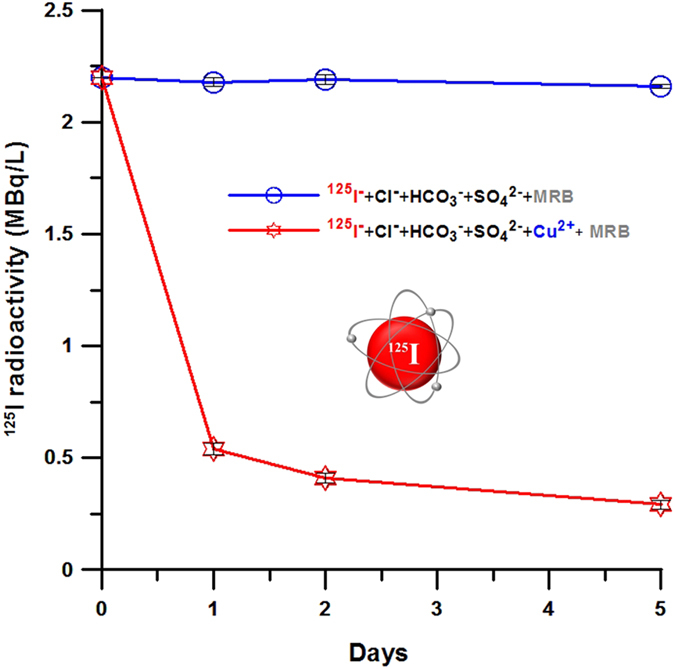
Practical performance for the reduction of high ^125^I radioactivity (>MBq/l) in anion-rich solutions. Aqueous radioiodine (^125^I) was effectively removed from the solution that was biostimulated with MRB and copper.

A large quantity of radioiodine with high radioactivity in water that contains competitive anions was targeted to be reduced by our system, and an actual test was successfully performed, as shown in Fig. 4, where strong ^125^I radioactivity (2.2 MBq/L) was decreased to a near non-detectable level for several days. In general, the radioactivity of iodine does not easily decrease in highly competitive anionic environments. Previous studies showed that some aqueous anions, such as chlorine and bicarbonate, act as strong competitors to I^−^16,29. In our microbial Cu-reducing system, however, the selective isolation and enrichment of radioactive ^125^I^−^ was not strongly influenced by Cl^−^ and HCO_3_^−^ or OH^−^species. More interestingly, most of the water-soluble radioiodine, such as ^125^I, was separable from the anion-rich water as a solid phase.

Various anion-exchanger and getter materials have been developed and evaluated for their capacities to sequester radioiodine in wastewater^15^,^16^,^17^,^18^. These materials generally have a good performance under acidic conditions, ranging from pH 2 to 6^30^,^31^. In higher pH ranges (e.g., pH ≥ 7.0), however, the anionic forms of iodine are repelled due to the negative charges resulting from growing effects of anionic OH^−^ and HCO_3_^−^ species. Furthermore, the capacity to remove iodine is restricted by innate properties, such as the specific surface area and adsorbing affinity. Recently, Ag^+^-doped materials^32^,^33^ have attracted attention as getter materials. They have shown better uptake for I^−^ compared to other substances, but they have several shortcomings for direct application to bulky wastewaters because of their high usage cost and limited capacity due to the monotonous chemical adsorption. Our new method that is proposed here, however, provides drastic cost reduction by using copper (Cu^2+^) ions, which are much cheaper than silver (Ag^+^). Moreover, the selective enrichment of iodide can further lower the disposal costs by reducing the radioactive waste volume.

In the past, there has been attempts to remove soluble iodide by using copper powder/plate plus azurite mineral^34^ or cuprous chloride (CuCl)^35^. However, the inorganic chemical method raised a problem to generate a large volume of waste due to the use of significant copper mass (solid or reagent) and final redundant discharge. When copper(I) was directly used as CuCl^35^, for example, considerable copper byproducts were produced, such as cuprous oxide (Cu_2_O) and copper sulphides. The major cause for this seems to be resulted from a condition of very fast and instant chemical reactions between copper(I) and soluble anions, which could prevent the copper(I) from stably interacting with iodide alone. In contrast, the gradual copper reduction from Cu(II) to Cu(I) based on the microbial redox reaction can generate favourable conditions, where stepwise CuI nucleation and growth are feasible, leading to the continuous growth to microscale crystals.

The increasing release and recycling of radioiodine has recently become a subject of intense public concern due its negative impact on the global ecosystem^5^,^6^,^7^,^12^,^20^,^21^,^22^,^23^. Thus, stronger preventive measures must be developed. Conventional methods based on anion-exchange and adsorption cannot cope with the growing release of radioiodine. We suggest a new solution that uses a simple biostimulant way to skillfully isolate radioiodine in the form of a solid salt that is easily separable from wastewater as an insoluble and precipitable phase. This study reveals that natural metal-reducing bacteria can make the free iodic anions grow into large crystal forms by iodine enrichment via the bioreduction of copper. Through the iodine enrichment process, halide nuclei can obtain momentum for robust growth. The basis of our system is to strongly restrict the volatile mobilization of iodide by dramatically transforming the mobile phase to an immobile crystal. The merit of the crystallization is to fix the iodide in a structurally rigid framework, preventing I^−^ remobilization later on. For example, marshite (CuI), which is a native mineral that is naturally synthesized by iodine and copper, is found to be a stable solid phase that has been preserved for long geologic times in the field^36^,^37^. This strong natural evidence indicates that the microcrystallization of radioiodine is a promising isolation method because the immobile solid phase is secure and stable in natural environments.

## Methods

### Anaerobic bacteria culturing

The anaerobic mixed culture containing a sulphate-reducing bacterium (*Desulphosporosinus auripigmenti*) was isolated from a bentonite sample located in a sedimentary rock in Kyongju (KJ), Korea. The culture (KJ) was obtained from the bentonite by a series of enrichment for approximately one year. Details of the enrichment process are described elsewhere^38^. The KJ culture’s phylogenetic tree and iron sulphide formation are shown in Figs S3 and S4. The components of the microbial-growth medium (per litre of deionized water) were as follows: NH_4_Cl, 20 mM; K_2_HPO_4_, 3 mM; MgSO_4_, 18 mM; CaSO_4_, 8 mM; sodium citrate, 25 mM; sodium lactate, 30 mM; and yeast extract, 1.0 g. A 100-ml volume of this medium was dispensed in a 120-ml glass serum bottle. Then, the medium was purged with pure N_2_ gas for 40 min, sealed with a butyl rubber septum, and capped with an aluminum seal. After the serum bottle was autoclaved, 5% ferrous ammonium sulphate (2 ml) was aseptically added to the medium using a syringe and needle. The medium was inoculated with the KJ culture for growth at 30 °C.

### Aqueous iodide removal experiment

To study the selective iodide removal from anion-rich water, we anaerobically prepared various anion-rich solutions of Cl^−^, HCO_3_^−^, and SO_4_^2−^ in serum bottles. The selected anions are common in natural waters, and their concentrations were as follows (mM): sodium chloride, 1.0; sodium bicarbonate, 3.0; and sodium sulphate, 1.0. Prior to the addition of iodide into the solution (100 ml), an iodide stock solution (100 mM) was prepared by dissolving a known amount of powder NaI in distilled water. From the iodide stock solution, 1 mM sodium iodide was aseptically added to the serum bottles containing the above anions.

To perform a practical test using an iodine radioisotope, a stock solution of radioactive ^125^I (half-life: 60 days) was prepared at 185 MBq/l from a reagent of 5 mCi Na^125^I (Perkin Elmer, Inc.). From the radioactive stock solution, ^125^I was injected to achieve 2.2 MBq/l radioactivity. In the radiological experiment, stable iodide (Na^127^I) was supplied to the solution at less than 1 mM to prompt iodide nucleation in the early stage.

Finally, cells were injected into the serum bottles, providing approximately 0.75 mg/l cell protein to determine the microbial effect on iodide removal. Organic matter (10 mM of sodium lactate) was injected through a 0.2-μm filter into the solution as an electron donor. To investigate a potential binder for mobile iodide (I^−^) in the microbial redox system, reducible cationic copper was selected and provided as a special electron acceptor and counterion in the iodide-containing media at less than 1.0 mM of Cu(NO_3_)_2_·3H_2_O. For a control test, silver (Ag^+^) ions were selected and injected at a concentration of 1.0 mM as AgNO_3_. The overall solution pH was ~7.3. The prepared serum bottles were then placed in a rotary-shaker (120 rpm at 30 °C) in the dark for 10 days. Periodically, liquid samples were removed by syringe and needle through 0.2-μm cellulose acetate filters and were analysed for changes in the stable iodide (^127^I) and copper concentrations using inductively coupled plasma-mass spectroscopy (ICP-MS). The inoculated cells were frequently removed to track their variation with time. Prior to the analysis of their protein quantity using a UV-Vis spectrophotometer (Cary 300, Agilent Technologies) at 562 nm, the cells were pretreated using a Micro BCA^TM^ Protein Assay Kit (Thermo Scientific).

### Analytical techniques

In the radiological test (10 days), the radioactivity (MBq/l) of ^125^I in a liquid sample that was passed through a 0.2-μm filter was measured by a liquid scintillation counter (LSC, Perkin Elmer, Tri-Carb 2910 TM) in the range of 0 to 100 keV for 30 min. A diluted solution of the liquid sample (0.5 ml) and distilled water (4.5 ml) was mixed with 15 ml of a cocktail solution (Ultima Gold^TM^). The prepared solution was kept in a cold and dark place for at least 20 hours before the gamma-ray measurement by the LSC.

To confirm the chemistry and structure of the solid samples produced in the system, the precipitable products were collected by centrifugation (10,000 rpm for 3 min) and were investigated by spectroscopic and microscopic analysers. For the purpose of the analyses, the solid samples that were separated from the solution were frozen and freeze-dried for 48 hours (Bondiro, Ilshin Co.). X-ray photoelectron spectroscopy (XPS) was performed to analyse the oxidation state using an AXIS Ultra DLD (Kratos Inc.) system with monochromatic Al Kα (1486.7 eV) and a 150 W source. To identify the mineral species and crystallinity, X-ray diffraction (XRD) analysis was performed with a Bruker D8 Advance automatic horizontal goniometer diffractometer, which was equipped with a scintillation counter and a Cu X-ray tube operated at 40 kV/30 mA in continuous scan mode.

For the electron microscope observation, a field emission scanning electron microscope (FESEM; S-4700, Hitachi) was used to acquire the morphological and chemical information of the solid samples. For the SEM observation, the samples were uniformly sprayed onto a carbon tape pasted on the specimen holder. To avoid charging during observation, the samples were coated with a thin OsO_4_ (~10 nm) layer. Chemical analysis was performed using an energy dispersive X-ray spectrometer (EDS; EMAX, Horiba). High-resolution transmission electron microscopy (HRTEM) was conducted for the iodide solid sample. To microscopically examine the internal structure of the solid phase, the specimen was washed three times in deionized water and embedded in Caldofix resin. Then, the blocks were sectioned into 50–70-nm-thick pieces using an ultramicrotome to obtain cross-sections of the sample. The sections were mounted on 200-mesh nickel grids, which were coated with carbon-sputtered Formvar support film for the HRTEM observation. The samples were examined by a JEOL JEM 2100F high-resolution field emission TEM at 200 kV. Elemental analyses were performed using an Oxford EDS system equipped with a SiLi detector coupled to the HRTEM and analysed by ISIS software.

## Additional Information

**How to cite this article**: Lee, S. Y. *et al*. Microbial copper reduction method to scavenge anthropogenic radioiodine. *Sci. Rep.* **6**, 28113; doi: 10.1038/srep28113 (2016).

## Supplementary Material

Supplementary Information
