# Supplementary material for: Microbial copper reduction method to scavenge anthropogenic radioiodine
Source: Sci Rep. 2016 Jun 17;6:28113. doi: 10.1038/srep28113 (PMC4911603; doi:10.1038/srep28113)
Supplement: Supplementary Information [file srep28113-s1.pdf]

Supplementary Information

Microbial copper reduction method to scavenge anthropogenic radioiodine

Seung Yeop Lee[1], Ji Young Lee[1], Je Ho Min[2], Seung Soo Kim[1], Min Hoon Baik[1],

Sang Yong Chung[3], Minhee Lee[3] & Yongjae Lee[4]

[1]Korea Atomic Energy Research Institute (KAERI), Daejeon 34057, South Korea. [2]Korean Association for Radiation Application (KARA), Seoul 04790, South Korea. [3]Department of Earth & Environmental Sciences, Pukyong National University, Busan 48513, South Korea. [4]Department of Earth Sciences, Yonsei University, Seoul 03722, South Korea.

Correspondence and requests for materials should be addressed to S.Y.L. (email: seungylee@kaeri.re.kr)

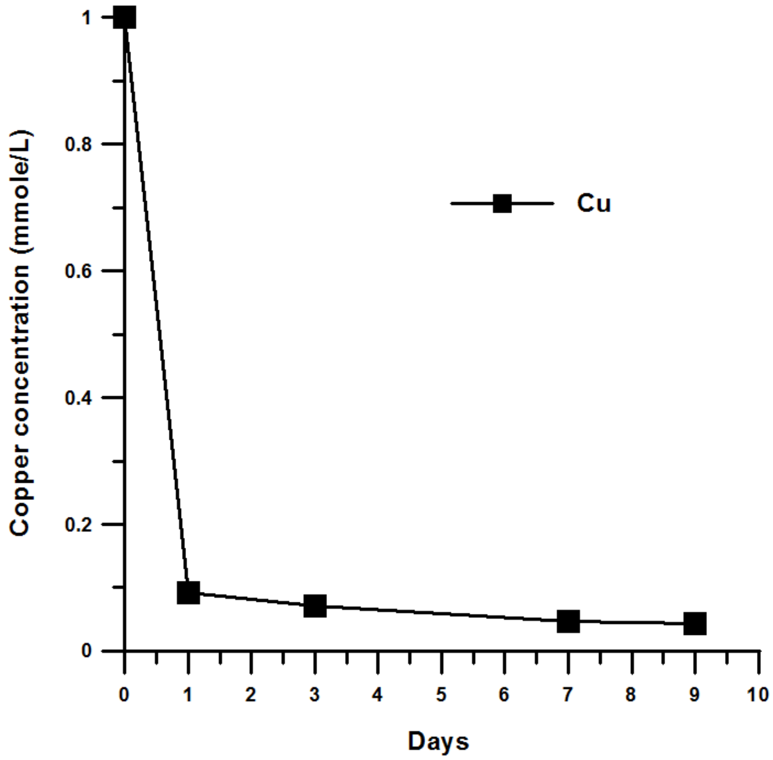

Figure S1. Decrease of aqueous copper(II) concentration during the microbial copper(II) reducing process in an anion-rich solution.

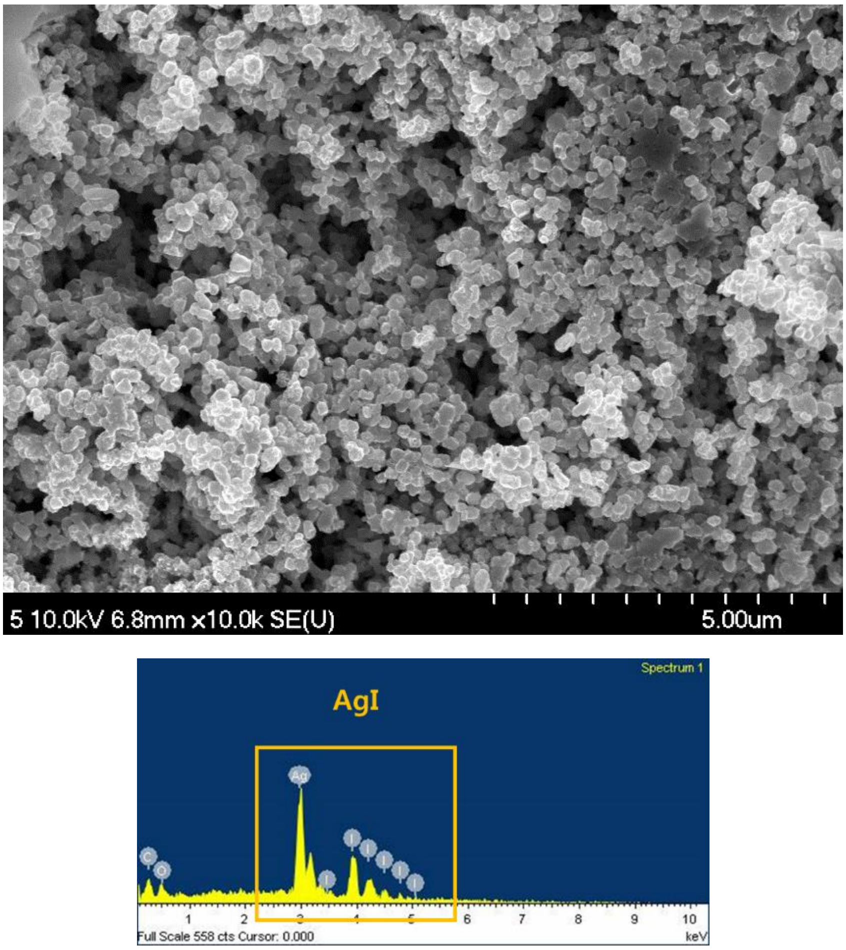

Figure S2. SEM image and EDS analysis for the sample of AgI solid that was precipitated from the anion-rich medium.

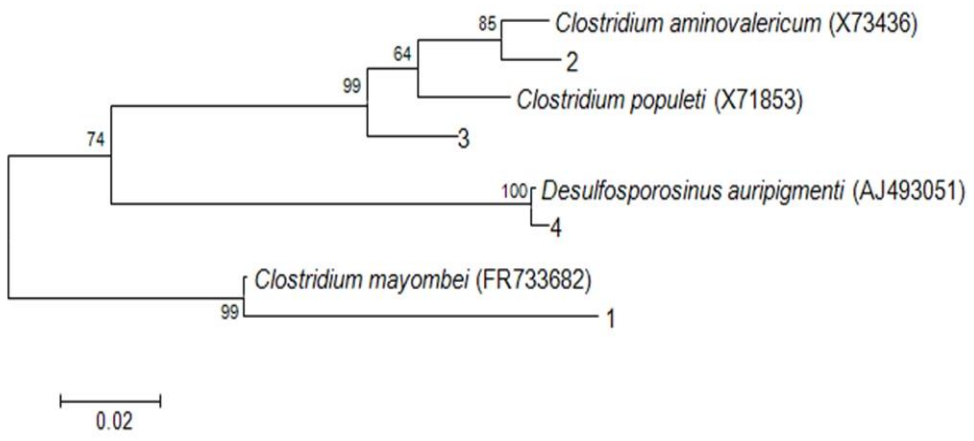

Figure S3. The phylogenetic tree based on 16S rRNA gene analysis for the KJ culture enriched from the Kyongju bentonite, Korea (ref. 38).

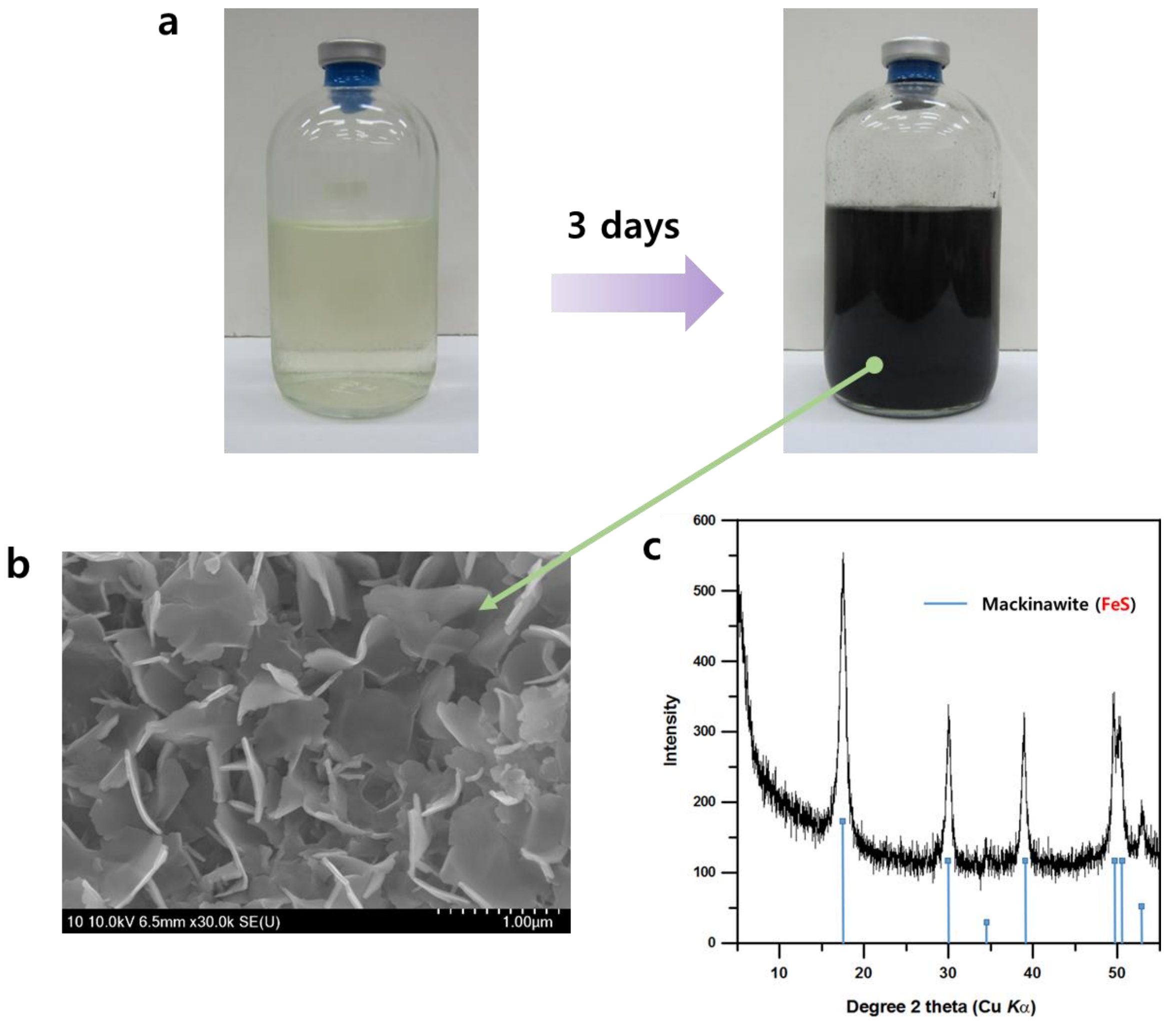

Figure S4. Biogenic iron sulfide formation from the KJ culture. (a) A color change in the iron and sulfate-containing medium from faint yellow to deep dark after the inoculation of KJ culture. (b) SEM image showing the microscale morphology of biogenic iron sulfide. (c) A characteristic XRD pattern of the iron sulfide (mackinawite; FeS).
